# Alignment-Free Wireless Charging of Smart Garments with Embroidered Coils

**DOI:** 10.3390/s21217372

**Published:** 2021-11-05

**Authors:** Chin-Wei Chang, Patrick Riehl, Jenshan Lin

**Affiliations:** 1 Department of Electrical and Computer Engineering, University of Florida, Gainesville, FL 32611, USA; changchinwei@ufl.edu; 2 Analog Devices, Wilmington, MA 01887, USA; Patrick.Riehl@analog.com

**Keywords:** wireless power transfer, wireless charging, inductive coupling, smart garment, embroidered coil, alignment-free wireless charging

## Abstract

Wireless power transfer (WPT) technologies have been adopted by many products. The capability of charging multiple devices and the design flexibility of charging coils make WPT a good solution for charging smart garments. The use of an embroidered receiver (RX) coil makes the smart garment more breathable and comfortable than using a flexible printed circuit board (FPCB). In order to charge smart garments as part of normal daily routines, two types of wireless-charging systems operating at 400 kHz have been designed. The one-to-one hanger system is desired to have a constant charging current despite misalignment so that users do not need to pay much attention when they hang the garment. For the one-to-multiple-drawer system, the power delivery ability must not change with multiple garments. Additionally, the system should be able to charge folded garments in most of the folding scenarios. This paper analyses the two WPT systems for charging smart garments and provides design approaches to meet the abovementioned goals. The wireless-charging hanger is able to charge a smart garment over a coupling variance $\frac{k_{max}}{k_{min}}=2$ with only 21% charging current variation. The wireless-charging drawer is able to charge a smart garment with at least 20 mA under most folding scenarios and three garments with stable power delivery ability.

## 1. Introduction

Nowadays, more and more people use wearable devices to monitor and improve their health conditions [1,2,3]. With the help of wearable devices and a mobile app, users can read real-time data or record data in devices for long-term monitoring. Although there are many wearable products available in the market, most of them are in the form of a watch or a fitness band. Wearable devices embedded in clothes are still not common in the market. Wearable devices embedded in clothes can provide a better way of sensing [4,5,6] because the sensor nodes can be distributed over more body areas than only the wrist. One of the challenges for smart garments is that the whole garment needs to be washable. A typical solution is to request users to remove the gadget that contains electronic parts before laundry [7]. However, such designs require extra attention and effort from users.

Another challenge of designing modern wearable devices is the power requirement. As the number of electronic devices owned by each person increases, how to conveniently charge all of them becomes a challenge. In traditional power cord charging, each device needs its own cord to charge. This becomes an issue when limited outlets are available, and causes wear on cords and connectors and possible damage due to overuse. WPT, on the other hand, can support charging multiple devices at the same time [8,9,10], which can alleviate the need for many outlets. Furthermore, since no charging connector is needed, the electronic parts can be fully sealed to make the whole garment washable. With proper design and optimization, a multi-coil receiver (RX) system can achieve high overall efficiency [11]. Moreover, such systems can be designed to fit into certain shapes for a better user experience in offices, homes, or vehicles [12,13].

The WPT RX coils typically fabricated with a printed circuit board (PCB) or copper wire are not suitable for wearable devices embedded in smart garments. Their fabrication methods are not compatible with the garment-manufacturing process. They are not flexible and not comfortable to wear. For wrist devices, RX coils fabricated with flexible PCB (FPCB) have been used. In [14], a charging station that complies with the daily storage routine is designed for smart watches. Although the FPCB is quite flexible and proved to be able to withstand 50 washing cycles [15], it is still not very suitable for smart garments because it is not breathable and will degrade user experience when it is worn on the body. 

Recently, an e-textile material coil, LEL, has been used to design RX coils for smart garments [16]. Although the coil is proved to be machine-washable and the garment and coil can be fabricated with the same process, it is still not breathable.

In our previous work [17], the properties of embroidered coils are studied. The results show that it can be used in wireless-charging systems designed for smart garments. However, the efficiency is only 14% for the hanger system and 10% for the drawer system. Additionally, the hanger system is sensitive to misalignment, and the charging current drops from 60 mA to 30 mA when the garment deviates from its neutral position, which is not uncommon in our daily life.

In this paper, wireless-charging systems with an embroidered coil array are further analyzed in detail and redesigned. The hanger system is optimized to have a more stable current over misalignment, and the drawer system is optimized to charge multiple RXs with a stable power delivery ability. The embroidered coil design and fabrication are also described in detail.

## 2. System Description

### 2.1. WPT Basics

The voltage and current relationships of a one-to-one wireless power transfer system can be written as in (1) [18]:

(1) $$\left[\begin{array}{c} V_{tx}\\ 0 \end{array}\right]=\left[\begin{array}{cc} j\omega L_{tx}+R_{tx} & j\omega M\\ j\omega M & Z_{rx}+R_{rx}+j\omega L_{rx} \end{array}\right]\left[\begin{array}{c} I_{tx}\\ I_{rx} \end{array}\right]$$

where *M* is the mutual inductance between TX and RX coil, $R_{tx}$ is the effective series resistance (ESR) of TX coil, $R_{rx}$ is the ESR of RX coil, and $Z_{rx}$ is the impedance of the load connected to the RX coil.

The impedance $Z_{in}$ seen at the TX coil can be written as:

(2) $$Z_{in}=j\omega L_{tx}+R_{tx}+Z_{ref,rx}$$

where $Z_{ref,rx}$ is the reflected impedance from the receiver side and can be written as [19,20]:

(3) $$Z_{ref,rx}=\frac{\omega^{2}M^{2}}{Z_{rx}+j\omega L_{rx}+R_{rx}}$$

### 2.2. Embroidered RX Coil and Receiver Circuit

**Figure 1:**
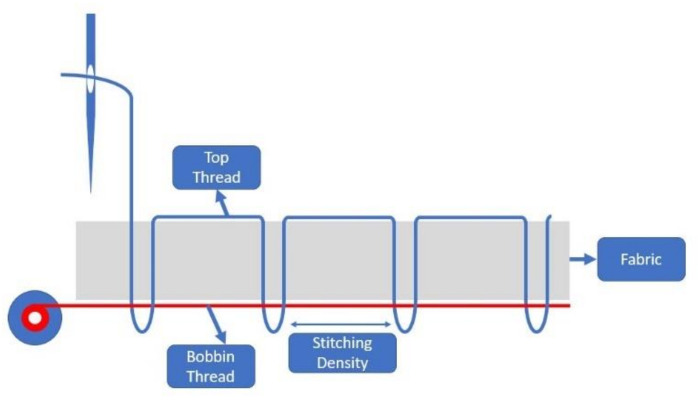
Embroidery process used in this work to fabricate RX coils on smart garments.

In this work, SewIY thread is used to fabricate the RX coils for smart garments due to its relatively high conductivity compared with other conductive threads and its insulated characteristic [21]. Figure 1 illustrates the embroidery process. Although the insulated thread is chosen in this work, it is not always a better choice than non-insulated threads, especially when it comes to mass production. During the embroidering process, there are some factors that will terminate the process, such as shorts of threads, broken threads, or broken needles. In some situations, the threads must be cut to fix the error. If the insulated thread is being used, the connection at the breaking point might need to be made manually. On the other hand, with a non-insulated thread, the connection will be made naturally as long as they have contact with each other. This can be easily ensured by moving a few stitches backward so that the two parts will overlap.

Based on our previous work [17], the quality factor of the embroidered coil can be improved by parallel-embroidering multiple threads to form the trace. One advantage of an insulated thread is that it allows a narrower gap between turns since there will be no unwanted connection between each turn due to process variation. With a non-insulated thread, the gap between each turn needs to be large enough to prevent unwanted connection. This is especially significant when multiple threads are used in parallel, and the embroidering process of multiple threads will cause the gap between turns to be slightly smaller than the original design.

When the conductive thread is used as a bobbin thread, the stitching density can be increased since the bobbin thread is not required to go through the fabric. With higher stitching density, the conductive thread can be held tighter.

**Figure 2:**
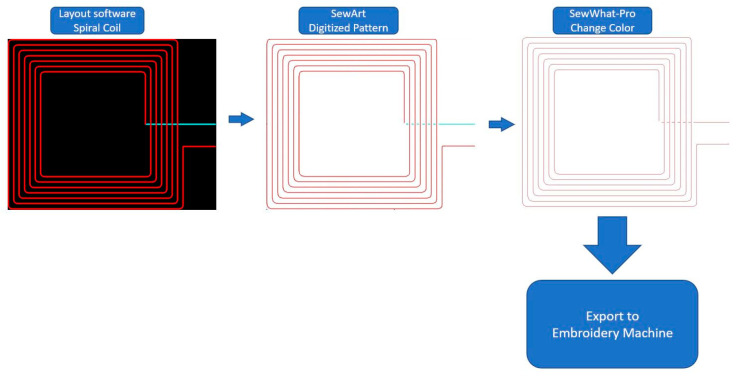
Process of creating embroidery file of an RX coil with a crossover stripe.

**Figure 3:**
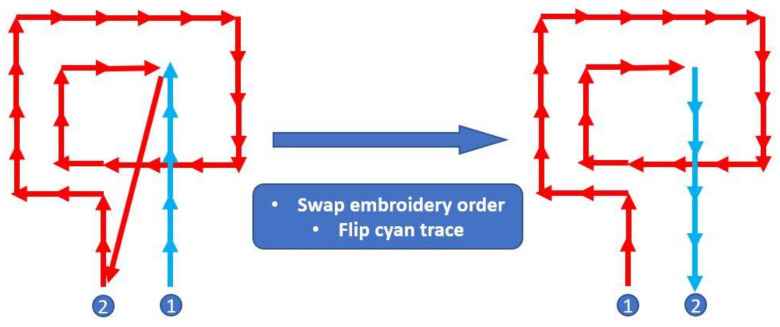
Correction of incorrect embroidery order and direction.

The embroidery machine used in this work is Janome Memory Craft 9850. Figure 2 shows the flow of creating an embroidery file of RX coil with crossover. The spiral coil pattern is created with layout software. It is worth noting that the color of the crossover (cyan stripe) needs to be different from the color of the coil. If the colors of the coil and crossover stripe are the same, the digitizer will see them as one object and will digitize it based on the algorithm instead of moving along the coil trace. A digitizer SewArt is used to convert the image file to an embroidery file. However, since the digitized pattern has two colors, the machine will cut the thread before using another color. This caused a problem since the SewIY thread is insulated, and connection needs to be made manually if the pattern is not completed at once. To avoid this scenario, an embroidery file editor SewWhat-Pro is used to adjust the stitching. The file editor can change the color of the thread used in the embroidery file and adjust the orders of stitching. Once the color is changed to the same color, the built-in simulator can be used to emulate the stitching to make sure it moves along the trace as expected. If the coil and crossover are made in the incorrect order and direction, as shown in Figure 3, this error can be fixed by swapping the order of the crossover stripe and coil and then flipping the crossover stripe.

**Figure 4:**
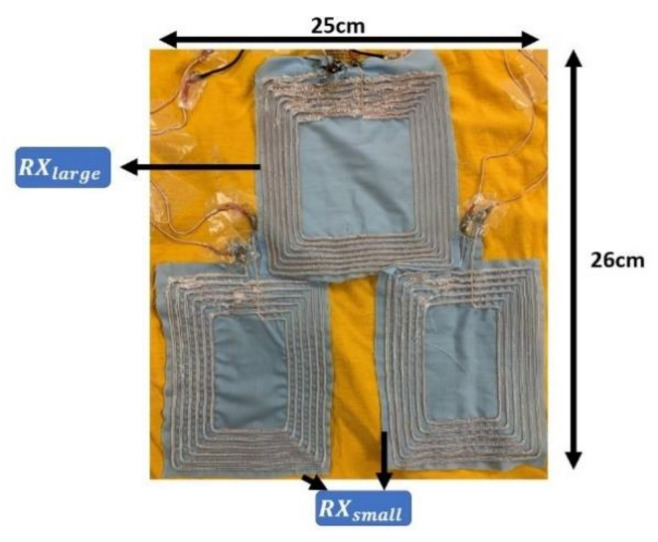
Embroidered RX coil array.

**Figure 5:**
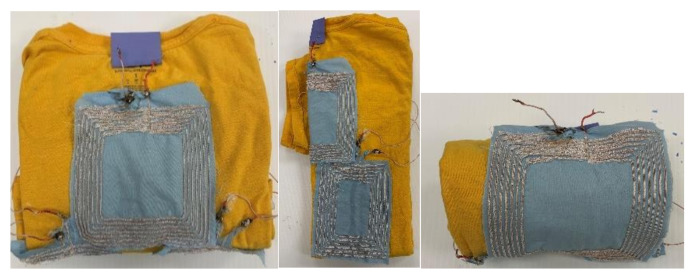
Embroidered RX coil array under different folding scenarios.

When drawers are used to organize clothes, it is inevitable that the clothes need to be folded. The three-coil array design shown in Figure 4 is able to avoid the scenarios when all the coils are folded. Figure 5 shows the RX coil array under some common folding scenarios. As it can be seen, in most scenarios, there will be at least one RX coil unfolded to receive power from TX. The received voltage from each RX coil in the array is rectified separately to avoid the case in which coils with opposite polarity due to folding induce voltages with 180° phase difference. Table 1 shows the properties of individual coils in the three-coil array. The self-resonant frequency (SRF) of all coils is 10 MHz, which is 25 times the operating frequency (400 kHz) of the designed systems.

**Figure 6:**
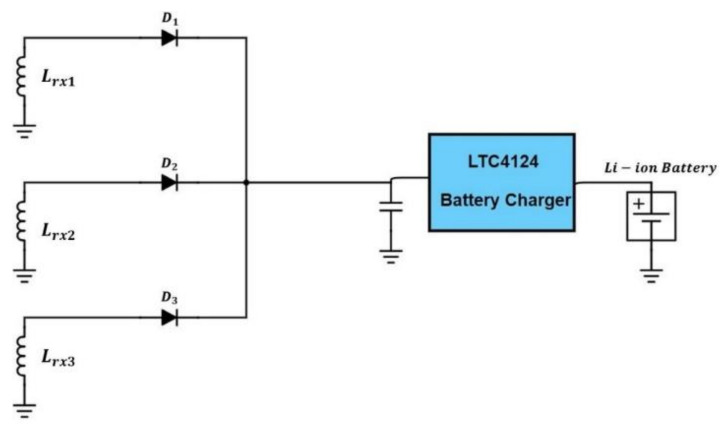
Circuit diagram of the RX coil array for smart garment.

**Figure 7:**
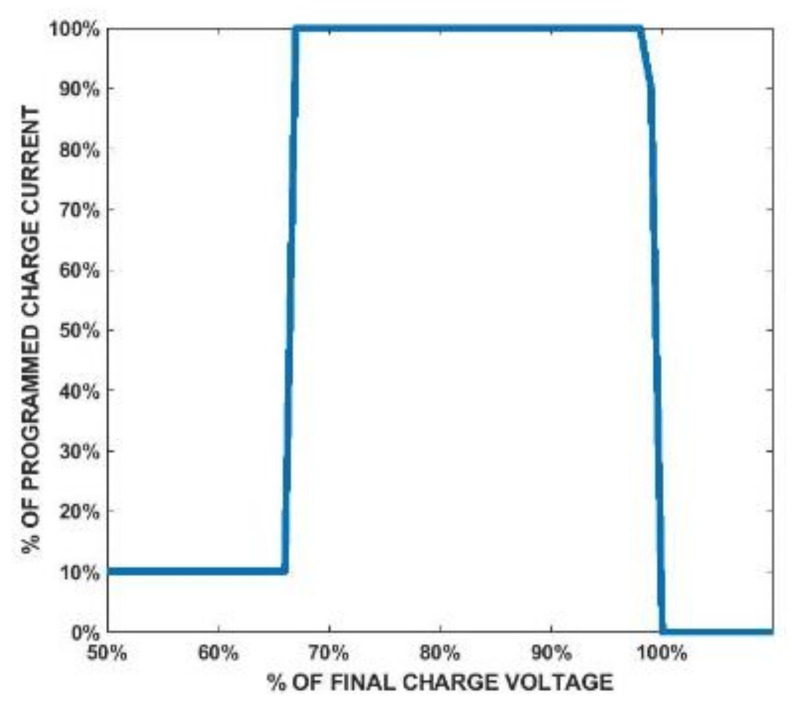
Charging profile of LTC4124.

Figure 6 shows the schematic of the RX circuit. It includes an RX coil array consisting of three coils, rectifying diodes, an LTC4124 battery charging module, and a Li-ion battery. Figure 7 is the charging profile of LTC4124. It is a module designed for a wireless-charging system and can charge a Li-ion battery up to 4.35 V with a maximum charging current of 100 mA. The module shunts excessive received power to maintain the $V_{cc}$ voltage at the cost of increasing temperature.

### 2.3. Wireless-Charging TX for Smart Garment Implementation

#### 2.3.1. Hanger Implementation

**Figure 8:**
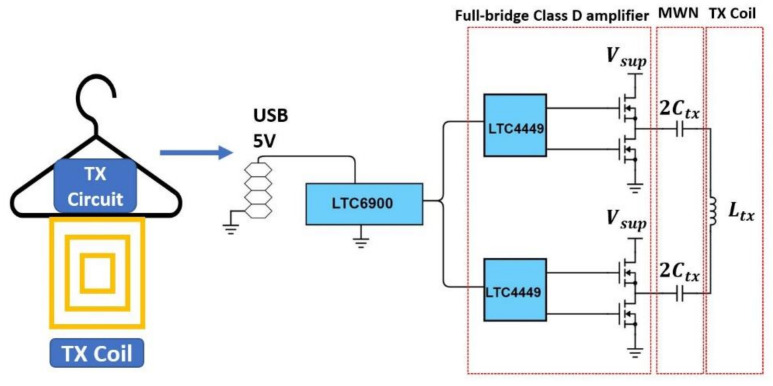
System implementation and schematic of the wireless-charging hanger.

**Figure 9:**
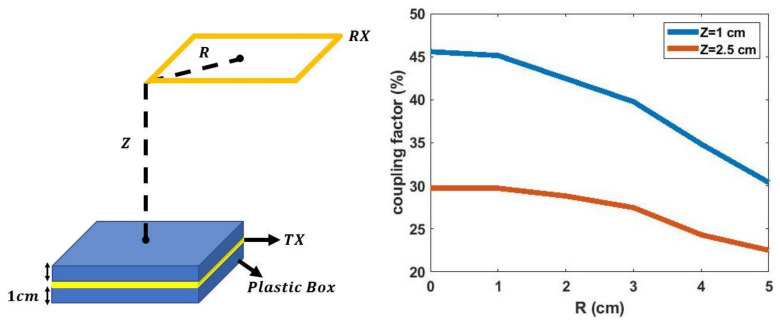
Measured coupling factor vs. alignment.

**Figure 10:**
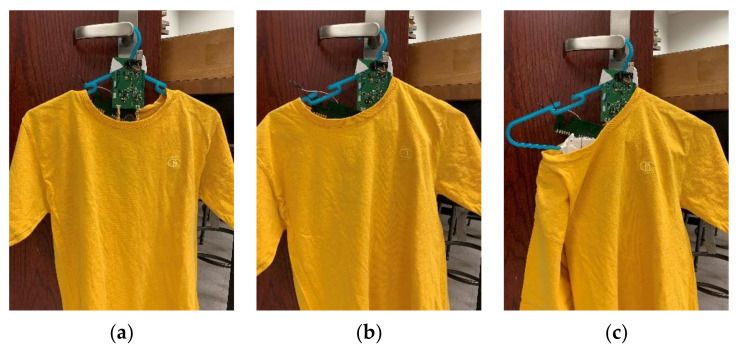
Three measured hanging scenarios: (**a**) neutral position; (**b**) garment slides to one arm; (**c**) garments falls from one arm.

Hangers are commonly used for organizing clothes. A WPT system implemented on a hanger allows integrating wireless charging with one’s daily routine. Figure 8 shows the implementation and schematic of the hanger WPT system. The TX circuitry and coil are placed on the hanger. The TX circuit includes a full-bridge Class-D amplifier, a matching network (MWN), and a TX coil. The matching capacitance $C_{tx}$ is replaced with two identical capacitors, each with a capacitance of $2C_{tx}$, to have a symmetrical circuit layout and thus reduce the common-mode emission from the PCB trace. When the RX coil array is used with the hanger TX, the large square coil will have a strong coupling with the TX coil and charge the battery. Table 2 shows the properties of the TX coil. To protect a user from moving too close to the TX coil, where the magnetic field may exceed the ICNIRP limit [22], the TX coil is encompassed by a plastic box to create a 2 cm separation from the TX coil. Figure 9 shows the measured coupling factor between the hanger TX coil and ${\mathrm{RX}}_{\mathrm{large}}$ within an estimated range of alignment tolerance. The range is the estimated misalignment and separation range between TX and RX when the garment is hung on a hanger. It is a cylinder with R=5 cm and Z=1.5 cm. Three hanging scenarios are also measured as in Figure 10, and the results are shown in Table 3. The result shows that the estimated range of alignment tolerance is able to cover most of the hanging scenarios.

#### 2.3.2. Drawer Implementation

**Figure 11:**
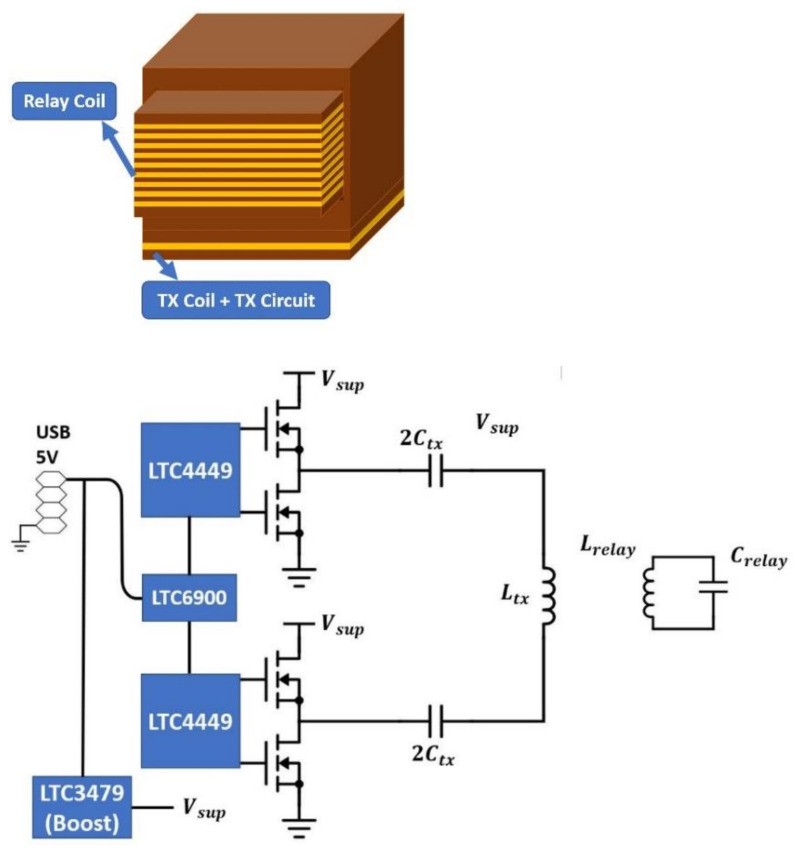
System implementation and schematic of the wireless-charging drawer.

Drawers are also commonly used for storing clothes. There are many studies on 3D wireless-charging systems with a box-shape transmitter. In [23], a TX coil cube is proposed, which is able to generate magnetic flux in all directions. In [24,25,26], four TX coils are used to create magnetic flux in two directions, and 3D receiver coils are designed to receive power regardless of their position and orientation. In [27], a TX coil with two turns is designed to surround the box to transmit power to the RX inside the box. In [28], a TX coil design using multiple high-Q resonant coils is used to power the headstage on animals inside the cage. Figure 11 shows the implementation and schematic of the drawer WPT system. A TX coil and a TX circuit are placed in the base of the drawer, and a relay coil surrounds the drawer. When we store garments in the drawer, the RX coils on the garments will mostly face up or down. Thus, the relay coil is designed to create a magnetic field in the z-direction. This implementation of the relay coil has two advantages. First, since the active components are placed only in the base and the relay coil is not physically connected to the TX circuit, the drawer with the relay coil can be moved freely. This makes the design implementation in furniture easier. Second, the impedance inversion between the TX coil and relay coil improves the stability of the system with respect to load. Considering that when there is no RX load in the drawer, the drawer system becomes a two-coil system and can be described by (1). If the relay is terminated with a capacitor and resonates at the operating frequency, the reflected impedance from (3) can be written as

(4) $$Z_{ref,relay}=\frac{\omega^{2}M_{tx-relay}^{2}}{R_{relay}}$$

where the denominator in (3) becomes $R_{relay}$ since the reactance of the terminated capacitor cancels out the reactance of the coil inductance. $R_{relay}$ is the ESR of the relay coil.

If the $\omega M_{tx-relay}$ is much larger than the ESR of the relay coil, $R_{relay}$, $Z_{ref,relay}$ will appear to be a large resistance, and thus the current drawn from the source can be reduced when there is no RX load. Table 4 shows the properties of TX and relay coils. The coupling factor between TX and relay coil ($k_{tx-relay}$) is 33%.

## 3. System Design

### 3.1. Optimization for Free Alignment of a Wireless-Charging Hanger

An FPCB is used in this work as the substrate of RX circuitry. As mentioned in the previous section, the charging module LTC4124 dissipates excessive received power through the ground. However, the small size and the characteristic of FPCB limit its capacity for heat dissipation. In order to avoid heating issues, it is desirable to have a constant charging current under all hanging scenarios.

**Figure 12:**
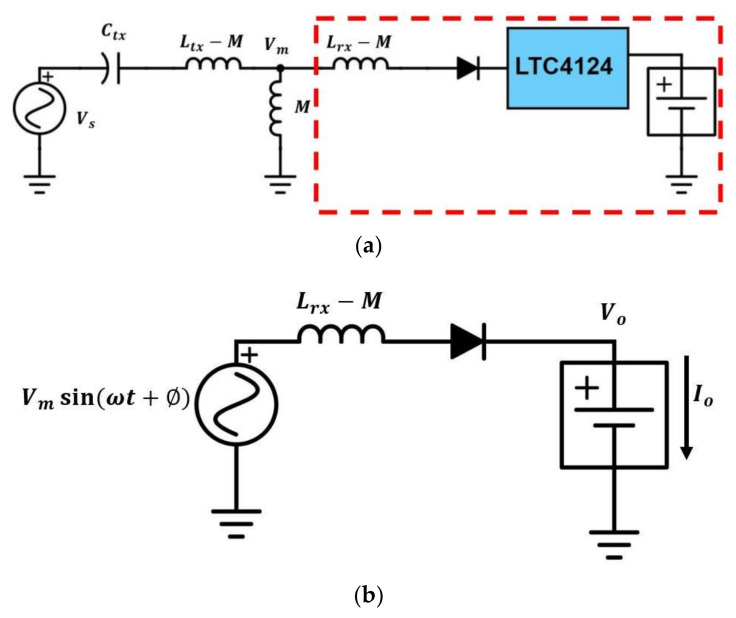
(**a**) Equivalent circuit for the wireless-charging hanger system; (**b**) equivalent circuit for the RX (dash block in (**a**)).

The equivalent circuit model for the wireless-charging hanger system is shown in Figure 12a. The coupling coil pair is replaced with a T-model. From [29], a constant-current (CC) output with a constant-voltage (CV) input can be achieved if the circuit satisfies the condition in (5). However, this technique is not applicable to this work since the current gain varies with mutual inductance.

(5) $$\omega =\frac{1}{\sqrt{L_{tx}C_{tx}}}$$

In the equivalent circuit model shown in Figure 12a, the receiver side in the dashed block can be considered as a low $\frac{di}{dt}$ Class-E rectifier [30,31] with a sinusoidal voltage source of amplitude $V_{m}$, a varying input inductance that equals $L_{rx}-M$, and a battery load as shown in Figure 12b. The Q of the Class-E rectifier can be defined as in (6):

(6) $$Q=\frac{R_{L}}{\omega \left(L_{rx}-M\right)}$$

where $R_{L}$ is the DC load resistance. From [30], $R_{L}$ can be expressed as in (7)

(7) $$R_{L}=\frac{V_{o}}{I_{o}}$$

In this case, $V_{o}$ is the voltage of battery and $I_{o}$ is the charging current.

*Q* can be expressed solely by duty cycle *D* of the rectifier as shown in (8):

(8) $$Q=\frac{\pi }{{\left(1-\pi D\cot\pi D\right)}^{2}}$$

The conduction angle of the rectifier ∅ can be solved by using (9):

(9) $$\tan\emptyset =\frac{1-\cos2\pi D}{2\pi D-\sin2\pi D}$$

The voltage gain of the rectifier $A_{v}$ can be defined as in (10):

(10) $$A_{v}=\frac{V_{o}}{V_{m}}=\sin\emptyset$$

From (10), the $V_{m}$ required to charge the load at voltage $V_{o}$ with charging current $I_{o}$ can be calculated with (11):

(11) $$V_{m}=\frac{V_{o}}{\sin\emptyset }$$

**Figure 13:**
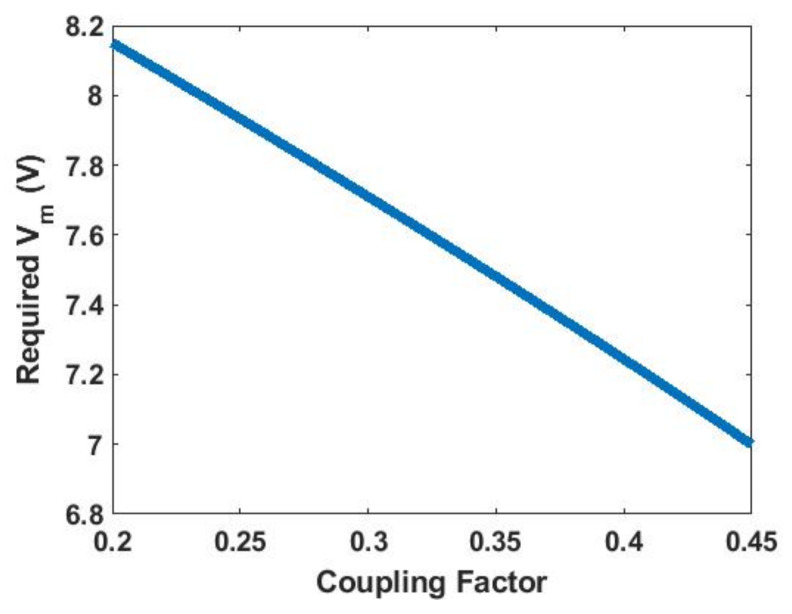
$V_{m}$ to achieve 100 mA charging current under different coupling factors.

With known $L_{tx}$, $L_{rx}$ and coupling factor range, the $V_{m}$ required to achieve a constant charging current can be plotted as in Figure 13.

**Figure 14:**
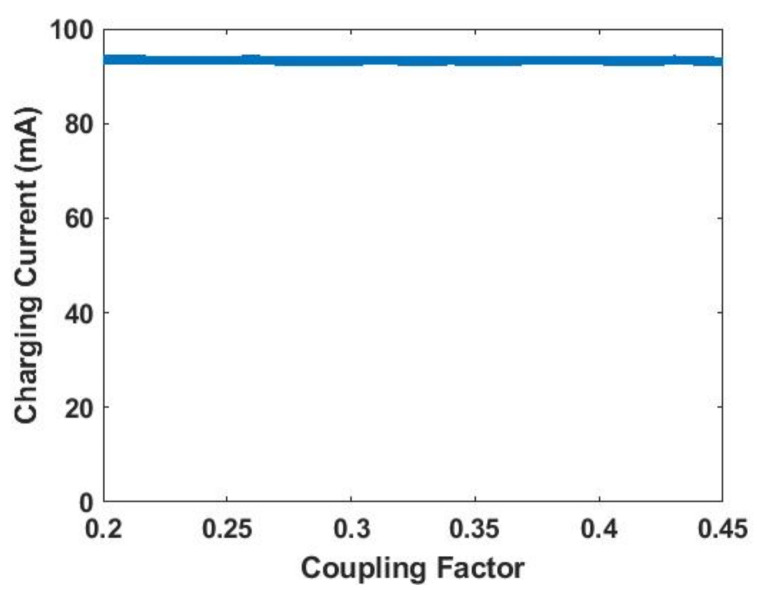
Simulated charging current vs. coupling factor.

Figure 14 shows the LTspice simulation result of Figure 12b using $V_{m}$ derived from (11). It can be seen that the charging current remains constant at 93 mA regardless of the coupling factor.

**Figure 15:**
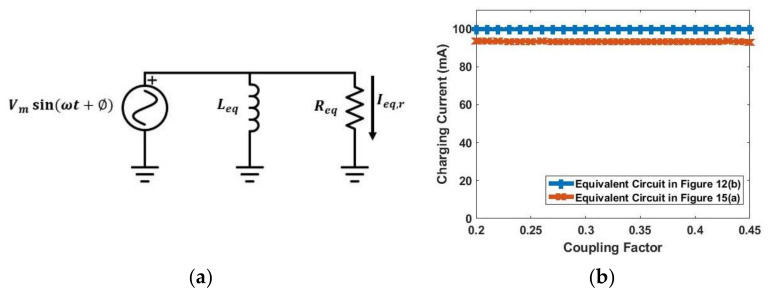
(**a**) Equivalent circuit model of Class-E rectifier; (**b**) simulation results using equivalent circuits in Figure 12b and Figure 15a.

The Class-E rectifier is replaced with its equivalent circuit [32], as shown in Figure 15a. The equivalent resistance $R_{eq}$ and the equivalent inductance $L_{eq}$ can be calculated using (12) and (13):

(12) $$R_{eq}=\frac{R_{L}}{2A_{v}^{2}}$$

(13) $$L_{eq}=\frac{L_{eff}}{E\left(D\right)}$$

where $E\left(D\right)$ can be written as in (14):

(14) $$E\left(D\right)=\frac{1}{2\pi }\left[2\pi D\cos2\pi D-\sin2\pi D\;\;\;\;\;\;\;\;\;\;\;\;\;\;\;\;\;\;\;\;\;+{\left(2\pi D\right)}^{2}\frac{\left(1-\cos2\pi D\right)\left(2\pi D-\sin2\pi D\right)}{{\left(2\pi D\right)}^{2}+2(1-\cos2\pi D-2\pi D\sin2\pi D)}\right]$$

Ignoring the losses from the diode and the ESR, the power received by the load should equal the power received by the equivalent resistor. This can be expressed as in (15):

(15) $$\frac{V_{m}^{2}}{2R_{eq}}=\frac{V_{o}^{2}}{R_{L}}$$

The charging current $I_{o}$ can be calculated as in (16):

(16) $$I_{o}=\frac{V_{m}}{2V_{o}}I_{eq,r}$$

where $I_{eq,r}$ is the current flowing through the equivalent resistor.

Figure 15b shows simulation results using schematics in Figure 12b and Figure 15a. It can be seen that Figure 15a models Figure 12b quite well.

**Figure 16:**
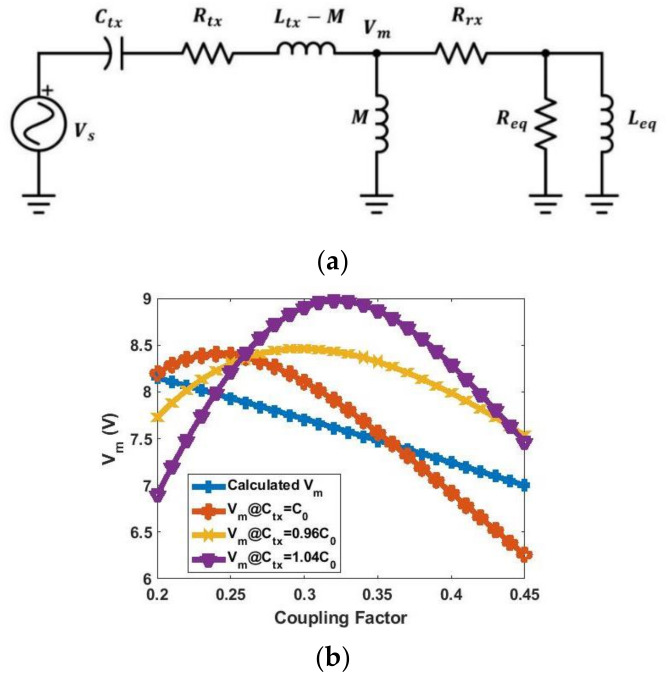
(**a**) Proposed equivalent circuit model of wireless-charging hanger system with RX replaced by equivalent Class-E rectifier circuit model; (**b**) simulated $V_{m}$ with different $C_{tx}$.

**Figure 17:**
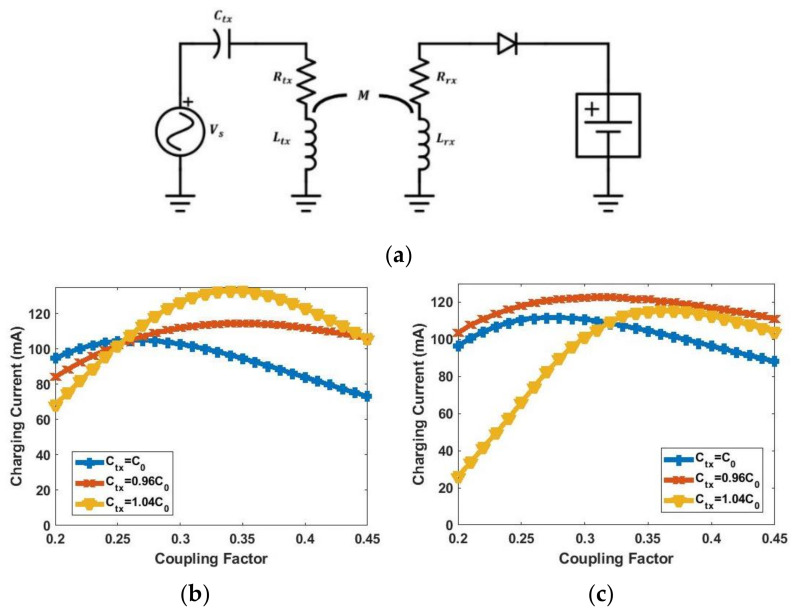
(**a**) Wireless-charging hanger system schematic without using Class-E rectifier circuit model; (**b**) simulated charging current using Figure 16a; (**c**) simulated charging current using Figure 17a.

Figure 16a shows the new equivalent circuit model of the wireless-charging hanger system. The ESRs of the two coils are included in this simulation. Based on the previous analysis, the optimum TX capacitance $C_{tx}$ is chosen to obtain $V_{m}$ derived previously. $V_{m}$ with different values of $C_{tx}$ is calculated based on the equivalent circuit model, and the results are shown in Figure 16b. Here, $C_{0}$ is the capacitance that resonates with $L_{tx}$. It can be seen that either $V_{m}$ when $C_{tx}=C_{0}$ or $C_{tx}=0.96C_{0}$ is close to the calculated $V_{m}$. Figure 17a shows the equivalent circuit model of the hanger system with mutual coupling and non-linear components. The charging current of the schematic from Figure 16a and Figure 17a are simulated and shown in Figure 17b,c. The results show that the non-linear model in Figure 17a can be approached with the linear model in Figure 16a. Additionally, both $C_{tx}=0.96C_{0}$ and $C_{tx}=C_{0}$ have relatively constant current over the coupling range. Table 5 summarizes the simulated charging current variation with differently matching $C_{tx}$ from Figure 17a.

### 3.2. Load-Independent Wireless-Charging Drawer

**Figure 18:**
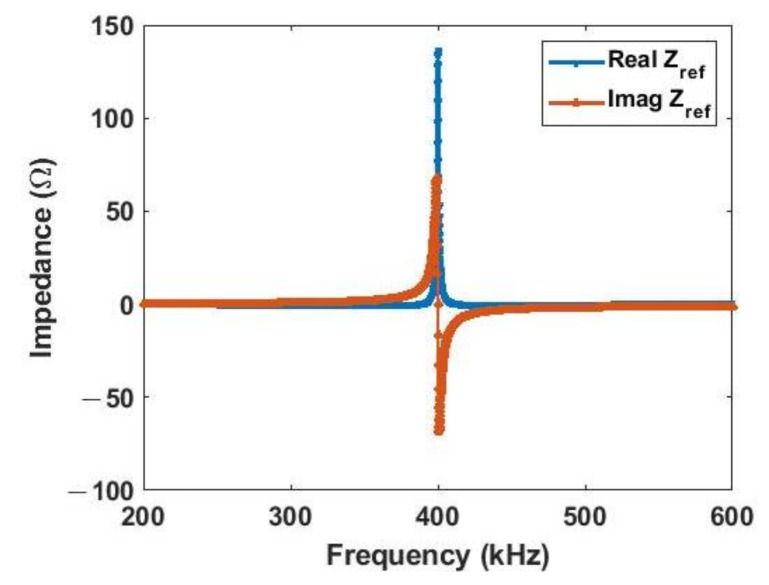
Simulated reflected impedance seen by TX coil without any RX load in drawer.

As mentioned in the previous section, if the relay coil is terminated with a capacitor that resonates with it at the operation frequency, the reflected impedance seen by the TX when there is no load in the drawer will be a large resistance. This can be shown by simulating the impedance, as shown in Figure 18. Thus, the current drawn from the power supply can be reduced when there is no load inside. The terminated capacitor is chosen to resonate with the inductance of the relay coil.

**Figure 19:**
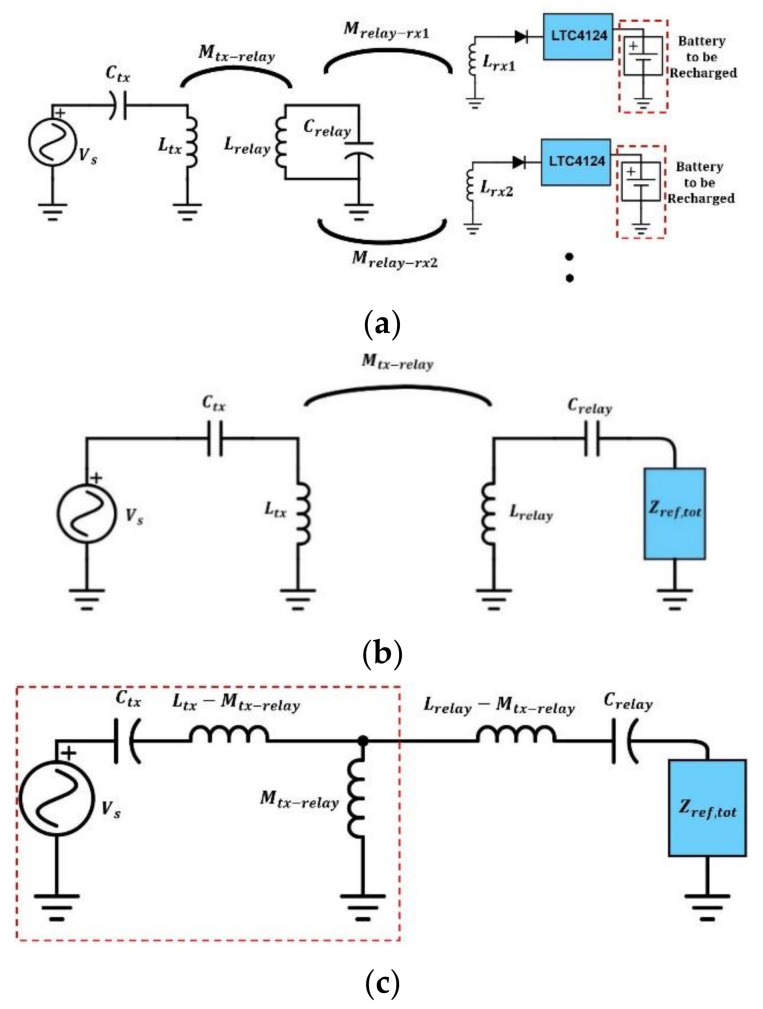
(**a**) Schematic of the wireless-charging drawer system with multiple RXs; (**b**) equivalent circuit model of the wireless-charging drawer system when the coupling between TX and RXs can be neglected; (**c**) equivalent circuit model with coil pair in (**b**) replaced with T-model.

Figure 19a shows the schematic of the drawer WPT system. Since the RXs are further separated from the TX coil and the inductance of the relay coil is designed to be much larger than the inductance of the TX coil, the mutual inductance between TX and RX coils can be neglected [33]. Usually, there will be multiple garments (multiple RX loads) stored in the drawer. It is desired that the power delivery ability of the drawer is not affected by additional loads. When multiple shirts are placed in the drawer, the RX coils on different shirts could overlap each other and might create a strong coupling between them. The coupling between RX coils, if strong, may reduce the charging current of each RX [34] and slow down the charging process. The probability of strong coupling can be reduced by using smaller RX coils or larger drawers. In the scope of this work, we consider the scenario when the RX coils on different shirts do not overlap each other so that the coupling between them is negligible. Neglecting the mutual coupling between RXs, the power received by each RX will be solely contributed by the relay coil. The *i*-th RX circuit can be modeled as an equivalent load $Z_{ref,rxi}$ connected in series with the relay coil in the equivalent circuit. The total load seen by the relay coil $Z_{ref,tot}$ will be the sum of all $Z_{ref,rxi}$ as in (17) [20].

(17) $$Z_{ref,tot}=\sum Z_{ref,rxi}$$

Under the assumption that the mutual coupling between RX coils is negligible, this can be proven by solving the voltage and current relation of such a system. For example, the voltage and current relationship of a scenario with two RXs can be described as:

(18) $$\left[\begin{array}{c} V_{relay}\\ 0\\ 0 \end{array}\right]=\left[\begin{array}{ccc} j\omega L_{relay} & j\omega M_{relay-rx1} & j\omega M_{relay-rx2}\\ j\omega M_{relay-rx1} & j\omega L_{rx1}+Z_{rx1} & 0\\ j\omega M_{relay-rx2} & 0 & j\omega L_{rx2}+Z_{rx2} \end{array}\right]\left[\begin{array}{c} I_{relay}\\ I_{rx1}\\ I_{rx2} \end{array}\right]$$

where $Z_{rxi}$ is the impedance connected to the *i*-th RX coil and $M_{relay-rxi}$ is the mutual inductance between relay coil and *i*-th RX coil. By solving (18), the impedance seen at the relay coil can be written as:

(19) $$Z_{relay}=j\omega L_{relay}+\frac{\omega^{2}M_{relay-rx1}^{2}}{j\omega L_{rx1}+Z_{rx1}}+\frac{\omega^{2}M_{relay-rx2}^{2}}{j\omega L_{rx2}+Z_{rx2}}$$

The last two terms on the right-hand side are the reflected impedance from the two RX coils, respectively. 

Based on the condition that the coupling between TX and RX coils is negligible [33], all the RXs inside the drawer can be seen as a load $Z_{ref,tot}$ to the relay coil, where $Z_{ref,tot}$ can be expressed as in (17) if the mutual coupling between RX coils can be neglected. The drawer system in Figure 19a can be reduced to a two-coil system, as shown in Figure 19b. The coupled TX and relay coils can then be replaced with a T-model [35], as shown in Figure 19c.

**Figure 20:**
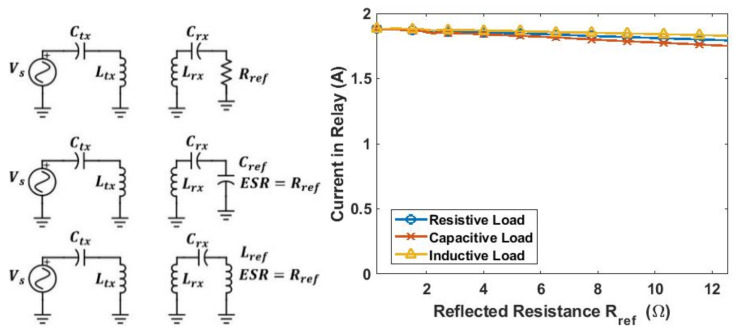
Simulated relay coil current under different loading conditions.

From [29], the part in the red dash block in Figure 19c can be seen as a CV to CC circuit if $C_{tx}$ is chosen to resonate with $L_{tx}$. Thus, by choosing $C_{tx}$ to resonate with $L_{tx}$, a constant current in the relay coil can be generated regardless of its load. A simulation is conducted in LTspice to prove this (Figure 20). The quality factor of both simulated capacitor $C_{ref}$ and inductor $L_{ref}$ are 1 and their ESR are both $R_{ref}$. The simulation result shows that the current in the relay coil remains stable regardless of the loading scenarios.

**Figure 21:**
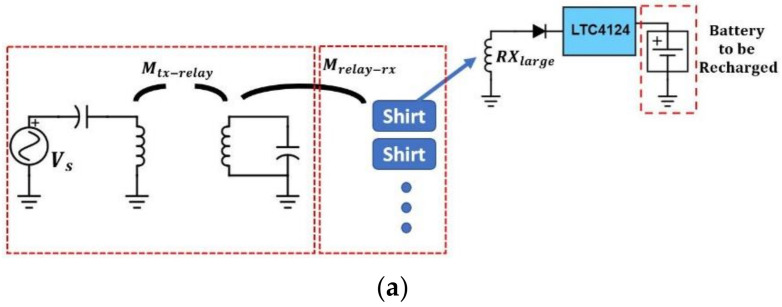

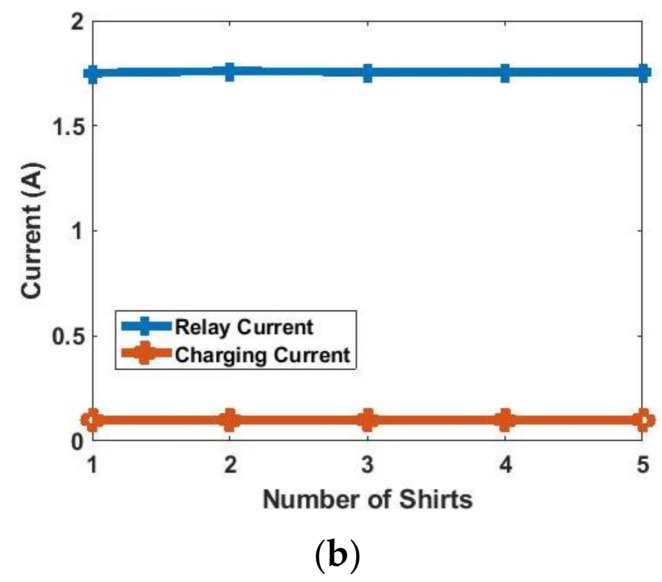
(**a**) Schematic of the wireless-charging drawer system with multiple shirts schematic; (**b**) LTspice simulation results.

Figure 21 shows the simulation of the wireless-charging drawer system with multiple shirts. As described previously, the mutual coupling between RXs is neglected in this simulation. The charging current shown in Figure 21b is the charging current of the first shirt. The result confirms that the current in the relay coil remains constant regardless of the load variation. Besides, as mentioned previously, since the mutual inductance between TX and RXs can be neglected, the relay coil behaves as a power source to the RXs. The charging current in Figure 21b shows that, when there is no mutual coupling between RXs, the charging current is solely determined by the current in the relay coil and is independent of the load of the relay coil (i.e., the number of shirts).

## 4. Measurement Results and Discussions

### 4.1. Measurement Setup and LTC4124 Module Test

**Figure 22:**
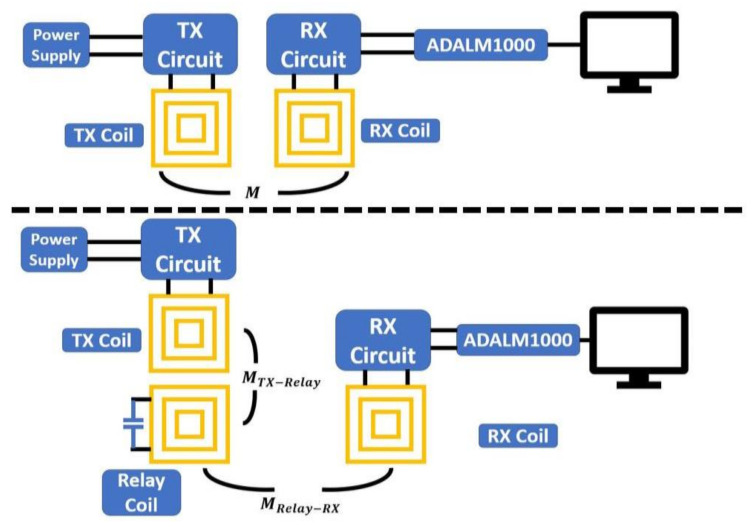
(**Top**) Measurement setup for the wireless-charging hanger system; (**bottom**) measurement setup for the wireless-charging drawer system.

**Figure 23:**
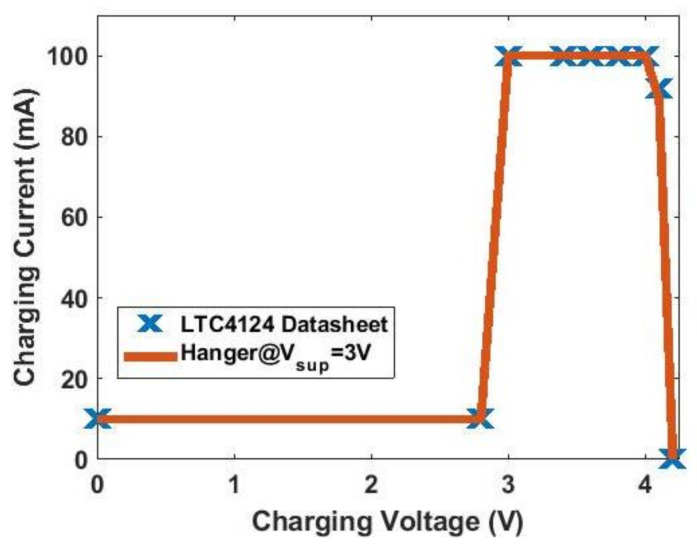
Wireless-charging hanger test with LTC4124.

In order to better emulate the situation when the battery is being charged, a source measure unit (SMU) ADALM1000 is used. The SMU is able to source a voltage or current and measure the current or voltage simultaneously on the same pin. The ADALM1000 can be controlled by an open-source software Pixelpulse 2 to adjust the voltage it sources. Figure 22 shows the measurement setups used in this section. The power of the system is provided by a power supply, so it will be easier to adjust. The TX circuit is connected to the TX coil with SMA connectors, and the battery pin is connected to the SMU with a cable. The LTC4124 module is tested by measuring the wireless-charging hanger system with varying charging voltage from 0 V to 4.2 V. The TX coil and RX coil in this measurement are aligned, and the separation between them in the z-direction is 1 cm. The measured coupling factor under this situation is 0.45. The charging current under different charging voltages is plotted and compared to the datasheet as shown in Figure 23. The supply DC voltage in this measurement is 3 V, and the current is 0.48 A. As shown in Figure 23, the measured charging current under different charging voltages complies with the datasheet. The DC-DC efficiency in this measurement setup is 27.8%. The result shows that the charging current starts to decrease when the charging voltage reaches above 4 V. In the following measurements, the charging voltage is set at 4 V to obtain the maximum charging power.

### 4.2. Wireless-Charging Hanger

**Figure 24:**
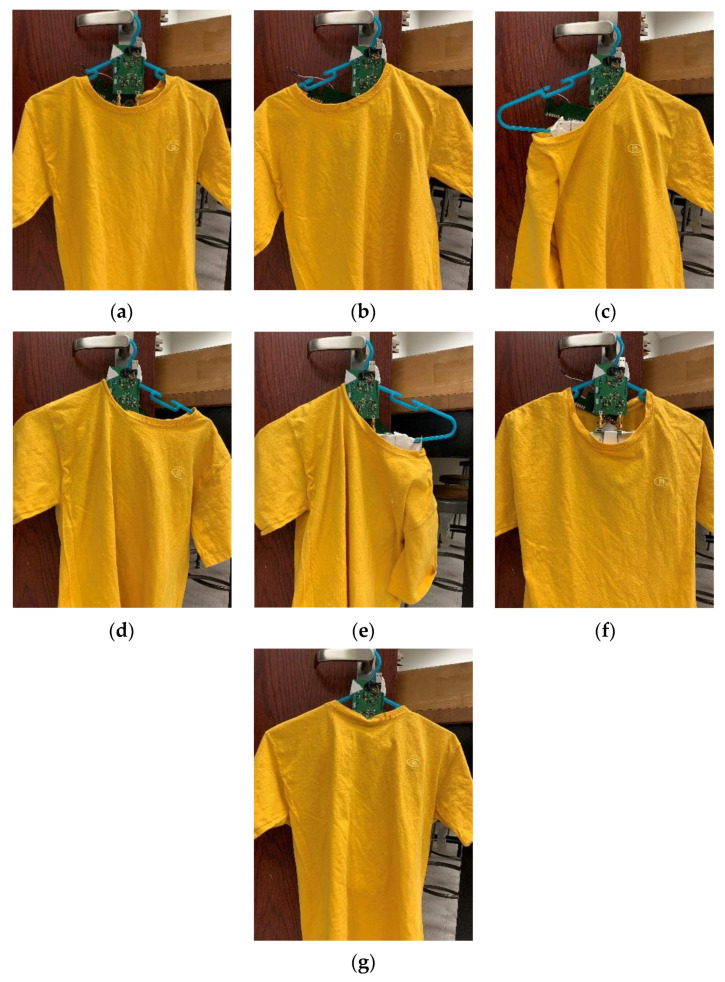

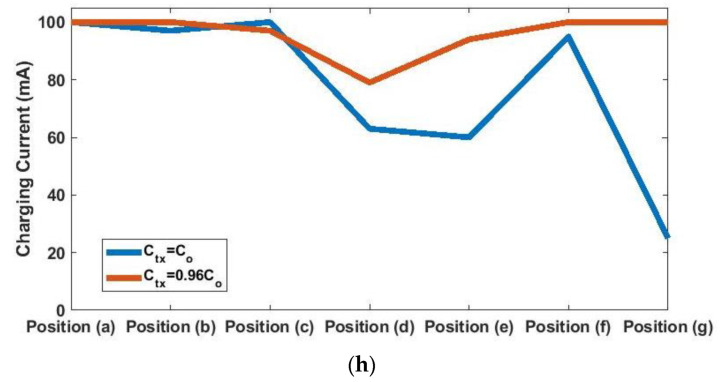
Measuring wireless-charging hanger system with different hanging scenarios: (**a**) garment in neutral position; (**b**) garment slides to left arm; (**c**) garment falls off left arm; (**d**) garment slides to right arm; (**e**) garment falls off right arm; (**f**) garment pulled from front side; (**g**) garment pulled from back side; (**h**) measured charging current with different $C_{tx}$.

Figure 24a–g show the seven hanging scenarios measured. Instead of using the three-coil RX array, only the large RX coil is used in this measurement to compare the results with the alignment-free concept described in the previous section. Figure 24c,e shows the worst hanging scenarios in which the shirt almost falls off the hanger. Figure 24h shows the measured results of these hanging scenarios. Two $C_{tx}$ values are tested in these measurements. The charging current varies only 21% regardless of the hanging scenarios when $C_{tx}=0.96C_{0}$, while it varies 75% when $C_{tx}=C_{0}$. The reason for the large charging current variation when $C_{tx}=C_{0}$ can be explained by the component variation and the parasitic capacitance of the RX coil trace. Referring to Figure 17c, the charging current when $C_{tx}=1.04C_{0}$ drops significantly when the coupling factor decreases, whereas the charging current remains relatively stable when $C_{tx}=C_{0}$. This implies that the system is quite sensitive to the value of $C_{tx}$ as the $C_{tx}$ in these two cases varies by only 4%. On the other hand, the charging current when $C_{tx}=0.96C_{0}$ is relatively stable over the coupling range. Thus, a $C_{tx}$ slightly smaller than $C_{0}$ can be used to compensate for the parasitic capacitance.

### 4.3. Wireless-Charging Drawer

**Figure 25:**
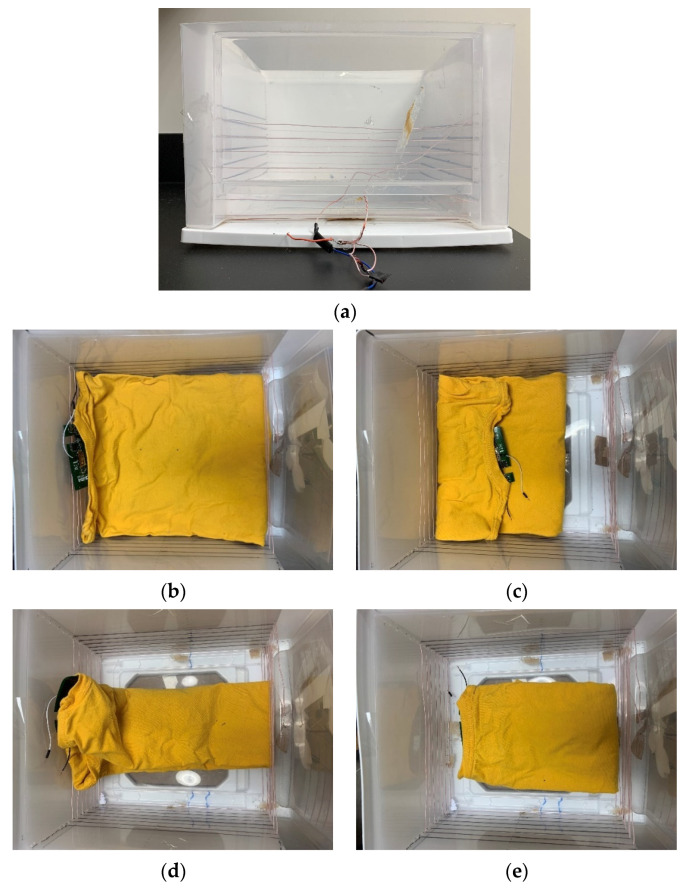

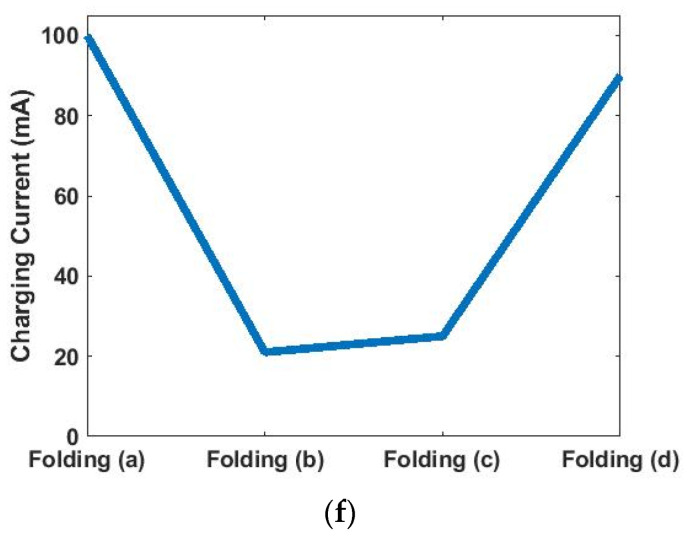
Measuring wireless-charging drawer system: (**a**) wireless charging drawer prototype; (**b**) no coil folded; (**c**) large coil folded and small coils close to drawer sidewall; (**d**) large coil folded and small coils in the center of drawer; (**e**) small coils folded and large coil in the center of drawer; (**f**) measured charging current under these four scenarios.

Two experiments are conducted to test the wireless-charging drawer system. Figure 25a shows the picture of a wireless-charging drawer. The TX coil is in the base, and the relay coil surrounds the drawer. Figure 25 shows the wireless-charging measurements of clothes with different folding scenarios. The folding scenarios can be grouped into three: no coil folded (b), large coil folded (c) and (d), and small coil folded (e), as shown in Figure 25. It can be seen from Figure 25e that the charging current in folding (d) in which the large coil is placed at the bottom center of the drawer where the weakest coupling occurs is above 80 mA. This means the charging current of such a system will be above 80 mA as long as the large coil is not folded. In folding (b) and folding (c), the two small coils together can provide at least 20 mA. Referring to Figure 5, the chance when only one small coil is not folded is very small under most common folding scenarios. This means that the wireless-charging drawer system can provide at least 20 mA charging current under most of the folding scenarios. The maximum DC-DC efficiency measured with one garment is 17.3%.

**Figure 26:**
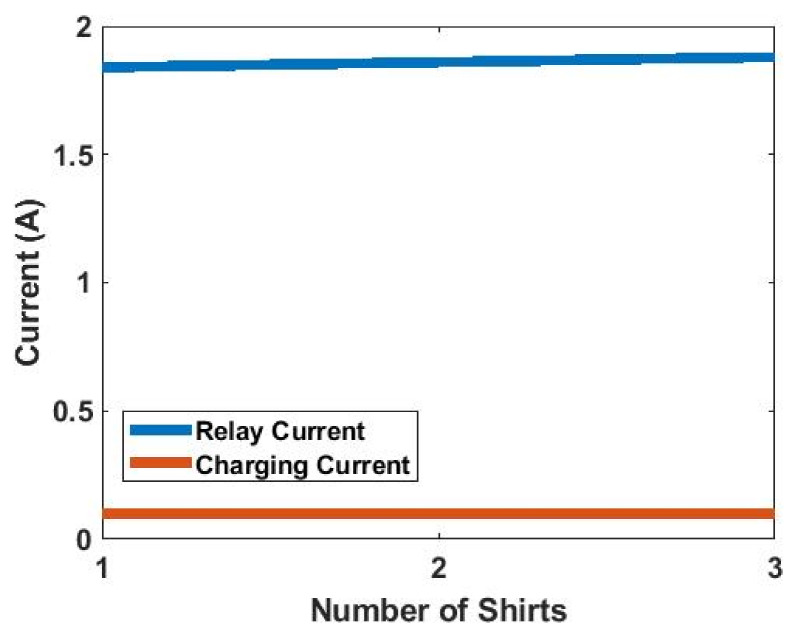
Measured charging current and relay coil current with different numbers of shirts.

The second experiment conducted for wireless-charging drawer is to test its capability of charging multiple garments with stable power delivery ability. A current probe is used to measure the current in the relay coil. The charging current of the first shirt is monitored when more shirts are added to the drawer. All the shirts are folded as in Figure 25b so that none of the RX coils are folded. As mentioned previously, the RX coils on different shirts might have significant mutual coupling if they overlap each other. Each shirt is rotated 90° and carefully placed to reduce the mutual coupling between them. Figure 26 shows the results of this measurement. It can be seen from Figure 26 that the charging current of the first shirt remains at 0.1 A when more shirts are added into the drawer. The relay current remains almost constant, which agrees with our previous analysis.

## 5. Conclusions

In this paper, two WPT systems and embroidered RX coils for smart garments are studied. For the wireless-charging hanger, the charging current varies only 21% over a coupling range of $\frac{k_{max}}{k_{min}}=2$ with a maximum DC–DC conversion efficiency of 27.8%. The measured results show that this design is able to charge a smart garment with a nearly constant current under most of the common hanging scenarios. For the wireless-charging drawer, the results show that the power delivery ability does not change when more shirts are added. At least a 20 mA charging current is guaranteed in the case when a large coil is folded, and an 80 mA charging current is guaranteed when a large coil is not folded. The maximum DC–DC efficiency with one shirt is 17.3% for the wireless-charging drawer.

## Figures and Tables

**Table 1:** Properties of individual coils in the embroidered RX coil array.

Coil	Inductance (μH)	Quality Factor	Dimension (cm × cm)
$RX_{large}$	9	8.35	13 × 13
$RX_{small}$	5.6	5.2	13 × 10

**Table 2:** Properties of the wireless-charging hanger’s TX coil.

	Inductance (μH)	Quality Factor	Dimension (cm)
TX coil	13.2	35	17 × 17

**Table 3:** Measured coupling factor of different hanging scenarios.

	Coupling Factor (%)	R (cm)	Z (cm)
$Hanging\;\left(a\right)$	37.35	0	1.5
$Hanging\;\left(b\right)$	24.22	6	1.5
$Hanging\;\left(c\right)$	22.82	6	2

**Table 4:** Properties of the wireless-charging drawer’s TX coil and relay coil.

	Inductance (μH)	Quality Factor	Dimension (cm)
TX coil	2	50	30 × 30
Relay coil	23.5	110	30 × 30 × 11

**Table 5:** Summary of simulation results of the proposed equivalent circuit model of wireless-charging hanger system.

$C_{tx}$	Maximum Charging Current $I_{ch,max}$ (mA)	Minimum Charging Current $I_{ch,min}$ (mA)	$\frac{I_{ch,min}}{I_{ch,max}}$
$C_{0}$	112	88	78.6%
$0.96C_{0}$	123	103	83.7%
$1.04C_{0}$	115	26	22.6%
